# Correction: van Lent et al. Kittens That Nurse 7 Weeks or Longer Are Less Likely to Become Overweight Adult Cats. *Animals* 2021, *11*, 3434

**DOI:** 10.3390/ani13060975

**Published:** 2023-03-08

**Authors:** Denise van Lent, Johannes C. M. Vernooij, Marcellina M. Stolting, Ronald Jan Corbee

**Affiliations:** 1 Lekker in je Vacht, Feline Behavioral Health and Welfare Centre, Secretaris Runsinkbrink 6, 2731 AG Benthuizen, The Netherlands; 2 Department of Population Health Sciences, Faculty of Veterinary Medicine, Utrecht University, Yalelaan 7, 3584 CL Utrecht, The Netherlands; 3 Cat Behaviour Consultancy, P.O. Box 11375, 1001 GJ Amsterdam, The Netherlands; 4 Department of Clinical Sciences of Companion Animals, Faculty of Veterinary Medicine, Utrecht University, Yalelaan 108, 3584 CM Utrecht, The Netherlands

The authors would like to make the following corrections to the publication [1]:

## Addition of an Author

**Marcellina M. Stolting** was not included as an author in the original publication [1]. The corrected Author Contributions Statement appears here.

**Author Contributions:** Conceptualization, D.v.L. and M.M.S.; Methodology, D.v.L. and M.M.S.; Data curation, J.C.M.V.; Validation, J.C.M.V.; Formal analysis, D.v.L. and J.C.M.V.; Investigation, D.v.L.; Project administration, D.v.L.; Resources, D.v.L.; Writing—original draft, D.v.L. and M.M.S.; Visualization, D.v.L., J.C.M.V. and R.J.C.; Writing—review & editing D.v.L., J.C.M.V. and R.J.C.; Supervision, R.J.C. All authors have read and agreed to the published version of the manuscript.

## Addition of an Affiliation

Accordingly, **Marcellina M. Stolting**’s affiliation and email is added (affiliation 3): Cat Behaviour Consultancy, P.O. Box 11375, 1001 GJ Amsterdam, The Netherlands; info@kattengedragstherapie.nl.

The authors apologize for any inconvenience caused and state that the scientific conclusions are unaffected. The original publication has also been updated.
