# Supplementary material for: Kittens That Nurse 7 Weeks or Longer Are Less Likely to Become Overweight Adult Cats
Source: Animals (Basel). 2021 Dec 1;11(12):3434. doi: 10.3390/ani11123434 (PMC8697871; doi:10.3390/ani11123434)
Supplement: Supplementary file 1 [file animals-11-03434-s001.zip › animals-1468844-supplementary.pdf]

Supplement material Table S1. Number of male and female cats per breed and Mean body fat percentage and Standard Deviation.

Breed	#Male	#Female	Total	Mean ± SD
Domestic shorthair	27	22	49	34.7 ± 9.14
Siamese	2	0	2	27.5 ± 3.28
Norwegian Forest Cat	4	5	9	26.7 ± 6.27
Ragdoll	1	0	1	37.2 ± na
Persian	0	1	1	25.9 ± na
Siberian	1	1	2	20.8 ± 1.51
Birman	1	0	1	14.4 ± na
Cornish Rex	1	0	1	26.4 ± na
British Shorthair	0	1	1	48.1 ± na
Russian Blue	2	0	2	26.8 ± 0.65

Abbreviations: SD= Standard Deviation, na = non applicable.

Table S2. Analysis of the cats (n=72) recruited through the veterinary database alone. Number (%) of cats per demographic factor against overweight status and unadjusted odds ratio with 95% CI*.

	Total		Overweight		No overweight			Confidence interval	
	n	%[1]	n	%[2]	n	%[2]	Odds Ratio	2.5%	97.5%
Total	72	100	37	51.4	35	48.6			
Length of suckling period (weeks)									
0 - 6	19	26.4	14	73.7	5	26.3	Ref		
7-11	24	33.3	12	50.0	12	50.0	0.36	0.09	1.3
12-16	21	29.2	6	28.6	15	71.4	0.14	0.03	0.54
17-24	8	11.1	4	50.0	4	50.0	0.36	0.06	2.0
Sex									
Male	42	58.3	23	54.8	19	45.2	Ref		
Female	30	41.7	14	46.7	16	53.3	1.3	0.49	3.2
Age (years)									
1.0 - 3.0	22	30.6	9	40.9	13	59.1	Ref		
3.5 - 5.0	20	27.8	7	35.0	13	65.0	0.78	0.22	2.7
5.5 - 7.0	13	18.1	10	76.9	3	23.1	4.8	1.1	26
7.5 – 12.0	17	23.6	10	58.8	7	41.2	2.1	0.58	7.8
Breed									
Purebred	21	29.2	4	19.0	17	81.0	Ref		
Domestic	51	70.8	32	62.7	19	37.3	7.2	2.3	28
Amount of food									
less or Prescribed	14	19.4	9	64.3	5	35.7	Ref		
More than prescribed + ad lib	58	80.6	27	46.6	31	53.4	0.48	0.13	1.6
Number of Meals per day									
1 – 2	38	52.8	22	57.9	16	42.1	Ref		
3 – 4	12	16.7	5	41.7	7	58.3	0.52	0.13	1.9
Ad lib	22	30.6	9	40.9	13	59.1	0.5	0.17	1.5
Playing with owner									
Daily	27	37.5	16	59.3	11	40.7	Ref		
Not daily	45	62.5	20	44.4	25	55.6	1.8	0.7	4.9
Free Outdoor access									
Yes	36	50.0	21	58.3	15	41.7	Ref		
No	36	50.0	15	41.7	21	58.3	0.51	0.73	2.8

Abbreviations: n= Number; Ref= reference category

[1] Percentage of category within the variable (column)

[2] Percentage of overweight status within the category of the variable (row)

Table S3. Final multivariable model* of the cats recruited through the veterinary database (n=72) with adjusted odds ratios and 95% confidence intervals for overweight.

Variable	Odds Ratio	Confidence interval 2.5%	97.5%
Length of suckling period (weeks)			
0–6	Ref		
7–11	0.48	0.11	1.8
12–16	0.13	0.03	0.56
17–24	0.31	0.04	2.0
Age (years)			
1.0–3.0	Ref		
3.5–5.0	0.85	0.22	3.2
5.5–7.0	5.0	1.02	30
7.5–12	3.0	0.72	14

* The full model started with variables: length of suckling period + age + sex + amount of food + number of daily meals per day + playing with owner + free roaming

Abbreviations: Ref = reference category
